# The Artificial Intelligence (AI) paradox

**DOI:** 10.6026/973206300221024

**Published:** 2026-02-28

**Authors:** Francesco Chiappelli, Quyen French, Allen Khakshooy

**Affiliations:** 1 Dental Group of Sherman Oaks, Sherman Oaks, CA 91403 & UCLA Center for the Health Sciences, Los Angeles, CA 90095; 2 Gratitude Dental, Fremont, CA 94539; 3 Internal Medicine at Valley Hospital Medical Center, Las Vegas, NV 89106

**Keywords:** Artificial Intelligence (AI), generative AI, high-performance computing (HPC), graphical processing units (GPUs), tensor protocol units (TPUs), machine learning (ML), deep learning (DP), ChatBots, deepfake technology, natural language processing (NLP), AI markup language (AIML), ChatGPT, AI-based clinical decision support systems (AI-CDSS)

## Abstract

The adoption of artificial intelligence (AI), from routine in every day usage to specialized interventions in dentistry and medicine, from personal needs to sophisticated business applications, has skyrocketed in high-income as well as in developing nations in the last decade. Inequalities remain however, and while AI utilization is practically worldwide, the divide between the northern and southern hemispheres is widening in terms of AI as the foundational infrastructure of modernity, and specifically of contemporary digital life. However, and to some extent paradoxically, the more AI is studied, developed and improved, the more we uncover its weaknesses, limitations, and, as some have argued, its inherent individual and societal dangers. AI generates information based on algorithms that are derived from factual data, which may, or may not have been verified by evidence-based science: information that carries the risk of being biased or fallacious at best, or old and invalidated by new research at worst. Critics of AI also observe its limits, such as in the context of basic human emotional and psychological-social skills. To be clear and as discussed in this writing, the more AI utilization grows and becomes more widespread, the more evident its limitations in reliability and validity become evident.

## Background:

## Artificial Intelligence (AI):

As a branch of computer science, Artificial intelligence (AI) is dedicated to creating systems capable of performing complex tasks (*e.g.*, reasoning, learning, computing, problem-solving, predicting and decision-making) attributed until now only to human intelligence. AI, as a field, consists of systems and models specialized in distinct tasks, which include, and are not limited to complex meta-data analysis, patterns identification, and prediction modeling. Current research and development in AI aims at refining its processes to be able to act and update itself increasingly autonomously, improving performance without explicit programming, in order ultimately to ensure reliability and validity of the output. Several AI models exist: each developed for a specific task or function, from complex cognitive elaborations, such as providing instantaneous written and voice translations of foreign phrases, to technical applications, such as self-driving cars, to sophisticated AI-driven decisions in critical government and commercial enterprises (*e.g.*, Palantir). AI is increasingly utilized in the health sciences as well, from biostatistical data analytics to medical and dental imaging and diagnostics, from individual searches of dental and medical information to personalized clinical intervention plans, from administrative processing to robotic surgery, from chairside dentistry and bedside medicine to tele-dentistry and tele-medicine, to cite only a few of the myriad of application of AI bioinformation technology in modern healthcare. AI models are constructed and organized by teams of scientists, or operators, into algorithmic layers that process, transform and utilize data.

## To be clear:

[1] input layers receive raw data (*e.g.*, text, images, meta-files of measurements and counts);

[2] covert, or hidden layers process the data, applying activation functions and non-linear transformations designed by the team of operators dedicated to developing the model; and

[3] Output layers that produce and generate the final operation (*e.g.*, prediction, classification, action), ultimately interpreted and evaluated by the operator team.

At the present state of the AI science, there are five principal domains of AI algorithms, that is to say five basic components of AI:

[1] Learning

[2] Reasoning

[3] Problem-solving

[4] Perception

[5] Language understanding in the case of cognitive applications, or practical applications in the case of mechanical actions, such as self-driving.

To be clear, the design, generation, testing and ultimately quality control of individual AI models constitute a scientific process that is planned, executed and evaluated by human intelligence based on the data and information available at the time of its execution. AI models increasingly also integrate an inherent ability to self-update (*e.g.*, generative AI) [1, 2-3]. However, in the case of evidence-based healthcare, which is grounded on the fundamental principle of generating the best available evidence through a systematic process of research synthesis and meta-analysis [4, 5-6], even generating and self-updating AI models of health information, diagnostics, and treatment interventions have difficulty keeping up with the fast rate of newly published scientific data daily or even on a monthly basis. In brief, AI is a computerized product of algorithms elaborated by human ingenuity, cognitive power, established knowledge and progress in scientific information and technological developments for the specific purpose of aiding certain human operations and functions. The AI infrastructure integrates the latest high-performance computing (HPC) technologies available, such as graphical processing units (GPUs) and tensor protocol units (TPUs), to power AI algorithms, including machine learning (ML) and its subset, deep learning (DP), that together define, characterize and underpin AI and its capabilities [7, 8]. Despite the superb advantages proffered by AI across various fields, including dentistry and medicine, as we have discussed in the context of immunology to fight pandemics [9] and despite concerted efforts to improve AI by incorporating self-updating potentialities, AI still suffers from serious limitations, particularly in the field of evidence-based clinical dentistry and medicine.

## AI benefits:

Unquestionably, the onset and development of AI in the previous decades has yielded and continues to proffer a great benefit to modern contemporary society. Originally conceived as a computerized technology to handle repetitive tasks faster and accurately, AI quickly developed into a most powerful tool to take in and to process unimaginably large inputs (*i.e.*, meta-data) to yield high-quality outputs in seconds. The efficient enhanced computational task automation process of AI is remarkable for its reliable analyses and predictions, "trusting" innovations, and overall efficacy especially in the domain of healthcare through the development of AI-information delivery platforms, or ChatBots integrated into websites, messaging apps (WhatsApp), and social media platforms (Facebook Messenger) [10].

## Dangers and limits of AI:

In healthcare, AI holds significant promise, as noted above, which could be simply summarized as enhancing health care delivery by providing more accurate diagnoses, personalized treatment plans, and efficient resource allocation. However, persistent concerns in AI utilization in dentistry and medicine remain, including serious ethical concerns, biases inherent to certain AI algorithms, lack of transparency in decision-making, potential compromises of patient data privacy, and covert, or not immediately apparent safety risks associated with AI implementation in clinical settings [11, 12- 13]. AI can create new and unique content such as images, text and music, as it can edit, correct, alter and falsify existing ones. Deepfake technology utilizes AI models to create images, videos, and/or audio that appears real but is fabricated. Deepfake outputs can have significant detrimental on education and student mental health [14], as well as patient education.

## The AI paradox:

One important concern about the use, over-use and perhaps misuse of AI is that it may lead individuals to rely less on their own cognitive abilities (*i.e.*, thinking, analyzing, reasoning, decision-making) and to depend more on what Chatbots, including Siri or Alexa, or more generally AI is suggesting. The more influenceable individuals could, by extension of this argument, become persuaded by the human-like nature of Chatbots, and even push the most vulnerable individuals toward a novel form of psychosis [15], or at least a sense of cognitive deficiency, deterioration and insufficiency, which has been termed "cognitive debt" [16]. In other words, paradoxically, AI that is supposed to aid psycho- cognitive tasks may actually negatively impact on our mental health and intellectual capacities: to be sure, an important hypothesis to investigate presently. AI is a tool developed in part to disseminate information. Yet, AI may often spread misinformation, which can be fast magnified by the nature of AI-powered software, which makes it easier to create and to spread it through a variety of deepfake technology platforms [17]. Paradoxically, therefore, the very new and attractive technology, which we trust to deliver up-to-date facts and truth, may indeed yield and systematically produce misleading information and misguided guidance.

At present, AI models are planned, designed and constructed as frameworks that usually handle well approximately 70% of repetitive, routine work of high-value activities, leaving to human intuition, creativity and cognition to address and resolve the remaining 30%. That is to say, roughly speaking, AI does the hard word (70%), leaving to humans the responsibility of the final judgment, and ethical decision-making (30%). But for how long? An important question not often asked, yet current bioinformatics research drives and pressures the field toward such refinements of AI protocols and models - including, for instance, sentience and morality [18]- to include, strengthen and perfect transferable high functioning life skills from communication, collaboration, critical thinking and ultimately creativity to AI. Inevitably, the current 70-30 ratio will be driven toward a 75-25 differential, and it might not be long - even an AI routine could be tasked to predict it - until we get to a 80-20 or even a 90-10 ratio. When computerize modules of AI handle 90% of a project - from its planning and designing, to its execution and data analysis, and ultimately draws the projects conclusion and best practices decisions - what will be the role of the human mind then? Will we not have given most, if not all the controls to AI? Paradoxically, when AI is let loose to drive a project from its inception to its conclusion, it might very well close and conclude it before we, its creator, are satisfied with its outputs and outcomes. Particularly relevant in this context are ChatBots, which are AI models, developed to be virtual social cognitive assistants. ChatBots, by their own design and intent, function with the purpose of using AI modalities, natural language processing (NLP), and machine learning, to understand user intent, to respond to queries promptly and precisely, and to adapt, update and evolve over time - *i.e.*, from early ELIZA of the 1960's to the Artificial Intelligence Markup Language (AIML)-based ALICE model of the 1990's, to the Siri, Alexa and other voice assistant systems of the 2010's, to our current context-aware, creative, and coherent generative AI Large Language Models (LLMs) *e.g.*, ChatGPT. Chatbots and other AI models are increasingly used in the clinic, and AI-based clinical decision support systems (AI-CDSS) are entrusted to enhance personalized medicine/dentistry to improve the efficiency of healthcare workers and altogether the efficacy of healthcare in general [19]. However, it is a fact that AI in general, and Chatbots in particular are not only presently limited as noted above, but they may also harbor some degree of deepfake reality, which could together seriously hamper their reliability and validity of clinical decisions. Therefore, while we increasingly seek to depend on AI and Chatbots in clinical decision making for improved patient care, these very tools may, paradoxically, carry hidden and covert threats to the very health and wellbeing of our patients. Where is the future pointing, if not to psycho-emotional and extended reality applications of AI? That is to say, AI in general and Chatbots in particular are expected to develop into aids toward enhancing emotional intelligence by integrating advanced multimodal capabilities, from integrating textual information, voice, and visuals. In dentistry and medicine, this step forward would translate into "conversational" AI-mediated care, an AI-driven dialogue on diagnosis-intervention [20]. Yet, paradoxically, the benefits of such developments may and will imply new and greater dangers of exposure to augmented false reality and, in general, more impactful deepfake damaging to the individual and the society at large before their benefits may become fully observable.

## Conclusion:

In conclusion and to paraphrase Einstein's well-known paradox, "the more I learn, the more I realize how much I don't know", the more we learn to use and to trust AI, the more we realize how much AI can be misused and misleading across the board including healthcare. Misguided AI-generated information - viz, misinformation, disinformation, fake news - is as pervasive a danger to individuals and to social groups as accurate AI-produced information can be beneficial and useful. This primary AI paradox has the following corollary: As we develop AI into a more powerful and broad-based instrument, and as AI takes on more and more the burden of thinking, doing, analyzing and evaluating to generate new questions and to conquer new horizons of science and knowledge, will the next generation of scientists, paradoxically, "not remember" that we created AI, how we created AI and for what purpose (*i.e.*, for the simple reason of sparing us the boredom of repetitive tasks)? Will scientists lose the critical thinking ability to know off-hand how to correct and redirect AI if and when needed? Will we then become, paradoxically, cognitively and factually subservient to AI - the very tool our cognitive abilities allowed us to construct in the first place to help and assist us in cognitive and factual tasks?
